# A low-cost wearable device for portable sequential compression therapy

**DOI:** 10.3389/frobt.2022.1012862

**Published:** 2022-11-14

**Authors:** Mark Schara, Mingde Zeng, Barclay Jumet, Daniel J. Preston

**Affiliations:** Department of Mechanical Engineering, Rice University, Houston, TX, United States

**Keywords:** stacked-sheet lamination, textiles, pouch motor, mechanotherapy, untethered, intermittent pneumatic compression (IPC)

## Abstract

In 2020, cardiovascular diseases resulted in 25% of unnatural deaths in the United States. Treatment with long-term administration of medication can adversely affect other organs, and surgeries such as coronary artery grafts are risky. Meanwhile, sequential compression therapy (SCT) offers a low-risk alternative, but is currently expensive and unwieldy, and often requires the patient to be immobilized during administration. Here, we present a low-cost wearable device to administer SCT, constructed using a stacked lamination fabrication approach. Expanding on concepts from the field of soft robotics, textile sheets are thermally bonded to form pneumatic actuators, which are controlled by an inconspicuous and tetherless electronic onboard supply of pressurized air. Our open-source, low-profile, and lightweight (140 g) device costs $62, less than one-third the cost the least expensive alternative and one-half the weight of lightest alternative approved by the US Food and Drug Administration (FDA), presenting the opportunity to more effectively provide SCT to socioeconomically disadvantaged individuals. Furthermore, our textile-stacking method, inspired by conventional fabrication methods from the apparel industry, along with the lightweight fabrics used, allows the device to be worn more comfortably than other SCT devices. By reducing physical and financial encumbrances, the device presented in this work may better enable patients to treat cardiovascular diseases and aid in recovery from cardiac surgeries.

## 1 Introduction

Cardiovascular disease is the top global cause of unnatural deaths, and has accounted for the largest increase in deaths since the year 2000 (World Health Organization, 2020). Globally, there were over 420 million cases of cardiovascular disease in 2015, with the top three most widespread and severe arterial diseases being deep vein thrombosis (DVT), peripheral artery disease (PAD), and coronary artery disease (CAD) (Roth et al., 2017). CAD, which is particularly deadly and more widespread than DVT and PAD, accounts for approximately one-third of all deaths in individuals over the age of 35 (Wilson and O’Donnell, 2017). In addition to the death toll, cardiovascular diseases can debilitate survivors and have consequently caused millions of people to suffer from residual issues long after acute treatment. As for DVT, within 1 year of diagnosis, between 17% and 50% of cases lead to post-thrombotic syndrome, which is a chronic and potentially disabling condition (Kesieme and Kesieme, 2011). Furthermore, PAD (the third-leading cause of death associated with cardiovascular morbidity worldwide) is becoming increasingly common, as the number of people diagnosed with PAD increased by 28.7% in low-to-middle-income countries and by 13.1% in high-income countries from 2000 to 2010 (Fowkes et al., 2013). The inverse correlation between socioeconomic status and risk of cardiovascular disease is reflected in higher-income countries as well, as evidenced by Steptoe et al. (2006). Their work corroborated that people of low socioeconomic status have a higher prevalence of risk factors for cardiovascular diseases. The causes of these cardiovascular diseases include age, smoking, systolic blood pressure, serum total cholesterol, diabetes mellitus, and a high body mass index (Celermajer et al., 2012). To avoid cardiovascular disease, most research suggests eating a balanced diet, being physically active, keeping a healthy weight, giving up smoking, reducing alcohol consumption, and keeping diabetes under control (Lonn and Yusuf, 1999).

Once a patient is diagnosed with cardiovascular disease, such as DVT, PAD, or CAD, the typical options for treatment to lower the risk of a significant cardiovascular event include 1) long-term use of medications, 2) high-risk revascularization surgeries, and 3) substantial lifestyle changes and structured exercise regimens (Gerhard-Herman et al., 2017). Sequential compression therapy (SCT) also functions to lower the risk of a significant cardiovascular event and is recommended alongside the typical treatment options. Additionally, SCT during walking or rehabilitative sessions is significantly more effective in reducing the risk for cardiovascular disease than either exercise or SCT alone (Sakai et al., 2021). Long-term medications are proven to be effective at addressing heart diseases, but they are often accompanied by side effects related to the patient’s liver or kidneys. Besides medications, coronary bypass surgery can be an effective treatment; however, it is a high-risk surgery and therefore is rarely performed, even on ideal candidates (Rodríguez-Olivares et al., 2018). Even for patients who undergo major open-heart surgeries, there remains a period of time following the operation where they are at high risk of a significant cardiovascular event before full recovery. Given the limitations of these options, SCT stands out as an enticing complementary or alternative approach. SCT is a form of moderated mechanotherapy that can both preemptively relieve the symptoms of heart diseases and render post-operative aid to patients as they recover after surgeries. Depending on the patient’s cardiovascular symptoms, different regimens of SCT could be used in a hospital or at home. A literature review of SCT studies found that most regimens of SCT included multiple sessions lasting 45 min each; however, there is a range of regimens which showed effectiveness with sessions lasting from 3 min to several hours (Phillips and Gordon, 2019). SCT shows promise as a treatment to cardiovascular diseases because it improves arterial blood flow in the limbs *via* mechanically pumping blood proximally, which leads to reductions in the risk factors for CAD (e.g., hypertension) by lessening the work needed by the heart to circulate blood (Labropoulos et al., 2005). SCT can also prevent PAD-caused critical limb ischemia from worsening into acute limb ischemia, which often leads to amputation (Labropoulos et al., 2005). Additionally, SCT is currently medically advised to help heal the heart if revascularization is performed to cure CAD (Maleti, 2016). Clinical data also suggest that after undergoing surgery to treat DVT, SCT lowers the risk of post-thrombotic syndrome by 60% (Gwozdz et al., 2020). Therefore, SCT not only functions as a treatment for cardiovascular diseases, but also offers significant value in recovering from a cardiovascular surgery.

**FIGURE 1:**
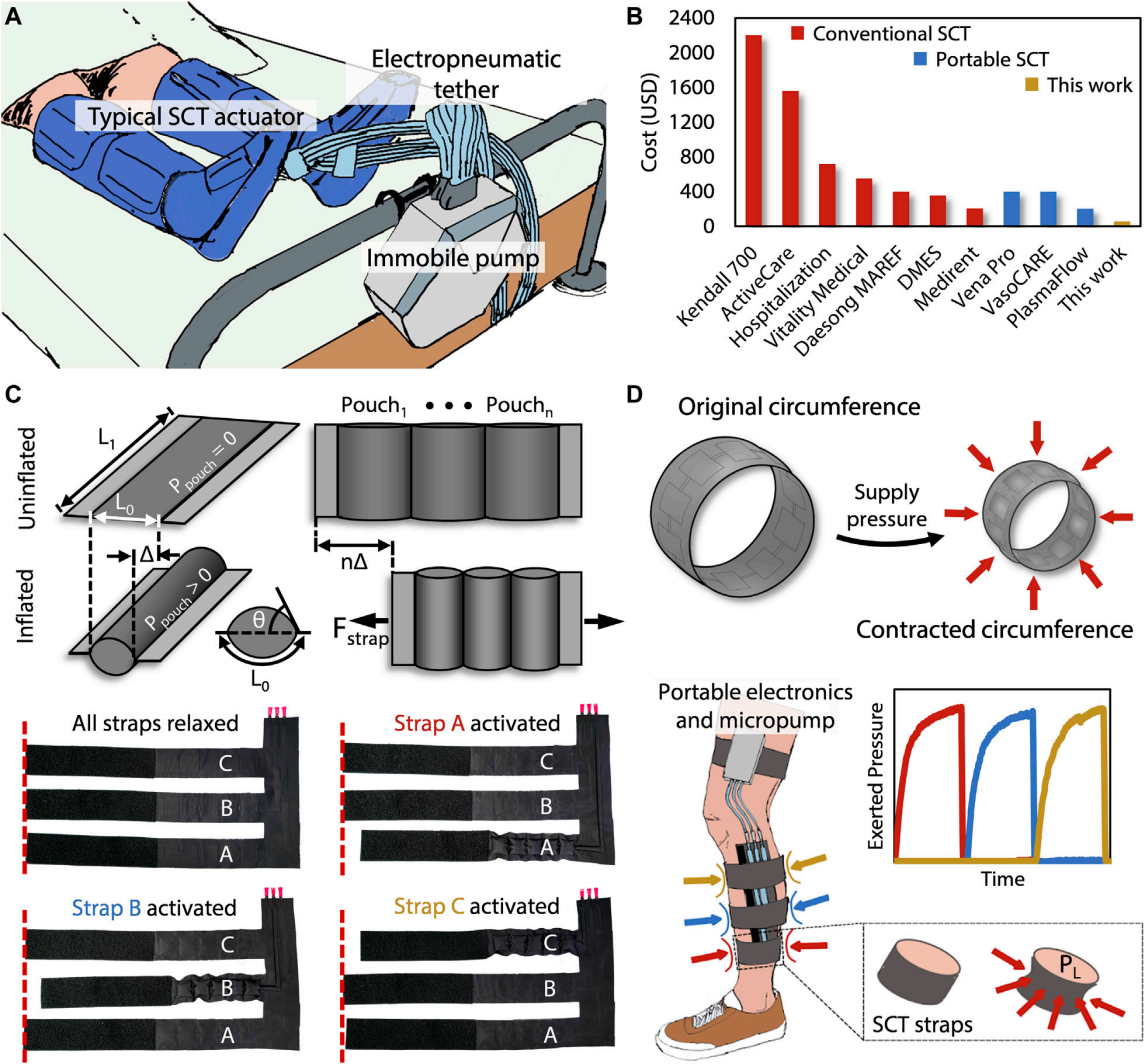
**(A)** Tethered and bulky conventional SCT devices limit the patient’s mobility. **(B)** Price comparisons are shown of the commercially FDA-approved SCT devices relative to our device. **(C)** Each strap of the presented device contains a pouch motor that applies a linear force (*F*_strap_) based on the dimensions *L*_0_ and *L*_1_, the pressure *P*_pouch_, and each constituent pouch’s tangential angle *(θ)* relative to the plane of the device. Each of these straps are sequentially activated, causing a contraction that constricts around the leg to apply an inward pressure. **(D)** The presented device performs sequential compression, using the same mechanism by which surface tension induces an analogous Laplace pressure (*P*_L_), starting at the distal end of the limb and progressing proximally toward the heart.

Unfortunately, conventional devices capable of performing SCT (Figure 1A) have numerous drawbacks that inhibit their effectiveness and accessibility as a treatment option for DVT, PAD, and CAD. These drawbacks include their bulkiness, immobility, and high cost. Often comprising cumbersome or tethered components, conventional SCT devices are heavy and uncomfortable, consequently limiting the user’s mobility and potentially shortening the duration of each session of SCT. Beyond the relatively superficial encumbrance of current devices, immobilization and shorter durations of SCT negatively impact the effectiveness of both the treatment of and recovery from DVT, PAD, and CAD (Zaleska et al., 2014). Besides their physically limiting designs, the high costs associated with SCT devices are a significant disadvantage, specifically due to the inverse socioeconomic correlation with cardiovascular diseases. As shown in Figure 1B, the cost of renting a conventional SCT device can range from $100 to $500 over the course of a 1-month treatment cycle, and buying one can cost over $1,000 (Figure 1B; Supplementary Table S1). Compounding these income-related disparities, people of low socioeconomic status have statistically higher rates of the risk factors for DVT, PAD, and CAD, including smoking, adverse lipid profiles, abdominal adiposity, and inflammatory markers (Steptoe et al., 2006). This fact, considered alongside the often-prohibitive cost of SCT, indicates that populations who need SCT the most are least likely to be able to afford it. Because SCT offers unique value in the treatment of ever-prevalent cardiovascular diseases, an affordable and more mobile SCT device is necessary.

In developing a portable and controllable SCT alternative, safety at the interface between humans and devices represents a critical concern that can be solved by inputs of risk-aware human models into robotic controllers (Kwon et al., 2020), the use of non-anthropomorphic designs (Tagliamonte et al., 2013), or making the devices intrinsically safe *via* soft robotic concepts. Soft robots lessen the safety risk associated with traditional (i.e., rigid) robots and are becoming especially beneficial in the design process for applications that regularly interface with humans. While textiles, a subset of sheet-based materials, are the least used category of soft materials in soft robotic components (Jumet et al., 2022a), they offer unique advantages in actuation (due to their ability to be designed for both strength and compliance) and in human-machine interfaces (because textiles are already ubiquitous in interfacing directly with humans). By leveraging their pliable geometry, the functions of sheet-based devices are diverse, ranging from oscillating actuators (Lee et al., 2022) to instability driven locomotion (Nagarkar et al., 2021). Going further, sheet-based devices made from textiles have been demonstrated in useful soft systems, such as functionally complete logic control (Rajappan et al., 2022) and power generation from walking (Shveda et al., 2022). Additionally, prior work in soft robotics has shown that textile-based wearable devices represent a promising solution for low-cost, untethered, and lightweight devices that apply forces to the body (Sanchez et al., 2021). Mechanotherapy is a well-suited application for soft roboticists because the intrinsic benefits of soft robotics, such as pliable human-device interfaces, are leveraged toward an important application: the promotion of healing muscle and bone tissue *via* mechanotransduction (Khan and Scott, 2009). For example, Payne et al. (2018) designed and tested a low-cost, low-profile, soft wearable device for muscle regeneration *via* mechanotherapy. More recently, Preston et al. (2019) demonstrated an inflatable mechanotherapeutic leg wrap as an application for a soft ring oscillator. Additionally, low-cost wearable devices, including a mechanotherapeutic device, were explored by Sanchez et al. (2020) as applications for smart thermally actuating textiles (STATs). These STATs were constructed by leveraging stacked fabrication of 2D sheets, to which textile devices lend themselves well. Beyond mechanotherapy, several authors have explored soft wearables as pneumatically actuated haptic devices that provide tactile (e.g., squeeze) cues to the body (Raitor, 2017; Agharese et al., 2018; Wu and Culbertson, 2019; Goetz et al., 2020; Jumet et al., 2022b). However, prior literature has not focused specifically on the development and characterization of a device to solve the problem of inaccessible SCT.

In this paper, we present a low-cost, soft, wearable device for SCT to provide comparable pressure delivery and functionality to the state of the art in a comfortable, mobile, low-profile, and inexpensive manner that consequently has a low impact on the user’s lifestyle. Differing in design from the targeted mechanotherapeutic devices which use one sleeve composed of a series of several large pouches that inflate inward to provide a compressive force (Payne et al., 2018), the presented SCT device has three individual straps, each composed of low-profile serial pouches called “pouch motors” (Mettam, 1962; Sanan et al., 2014; Yang et al., 2016) that wrap around the limb and constrict circumferentially to provide an inward pressure, in doing so providing the medically advised level of pressure and duration of compression necessary to conduct SCT. While more general compression sleeves have represented a common application space for soft robotic wearables in the literature (Payne et al., 2018; Preston et al., 2019; Sanchez et al., 2020; Jumet et al., 2022b), we instead focus in this work on the design and modeling of force transduced in the form of Laplace pressure (de Gennes et al., 2004), where circumferential force applied by a pouch motor is converted to normal pressure applied to the leg in our mechanotherapeutic device. As a potential solution to a common yet previously expensive problem, our device exhibits economic competitiveness at a cost of $62 and can be made from commercially available parts that are comfortable and (financially and physically) less inhibiting to the patient. An additional benefit for the presented mobile SCT is the small form factor of our device, which adds negligible weight and allows sessions of SCT to be easily started and stopped, which significantly improves quality of life. Even in cases where patients who are prescribed SCT struggle with getting in and out of bed and may require an attendant to put on the SCT device, our approach allows for a simpler and more comfortable approach than most. Furthermore, we demonstrate that our soft device performs comparably to existing commercial counterparts and can operate across the medically advised range of pressures for successful SCT.

## 2 Materials and methods

### 2.1 Fabrication

The device presented in this work relies on a two-dimensional (2D) stacked fabrication process. This process is advantageous due to its simplicity, which lends itself to inexpensive batch fabrication that is possible to perform in a do-it-yourself (DIY) manner, further enabling potential adoption by economically disadvantaged users. The 2D stacking technique enables the operational principle of the device: pouch motors (Mettam, 1962; Sanan et al., 2014; Yang et al., 2016). The pouch motor’s transition from planar rectangular sections to 3D cylindrical chambers *via* pneumatic inflation allows the transduction of applied pressure within the pouches to lateral tensile force by decreasing the effective length of the once-planar sections (Figure 1C). When the pouch motors are fashioned into straps which connect around the leg, the decrease in effective length of the straps becomes a decrease in the circumference of the strap that is now approximately circular. The Laplace pressure relationship indicates that circumferential force around a circular cross section induces a pressure directed radially inward, which is the essential mode of actuation for sequential compression therapy (Figure 1D).

**FIGURE 2:**
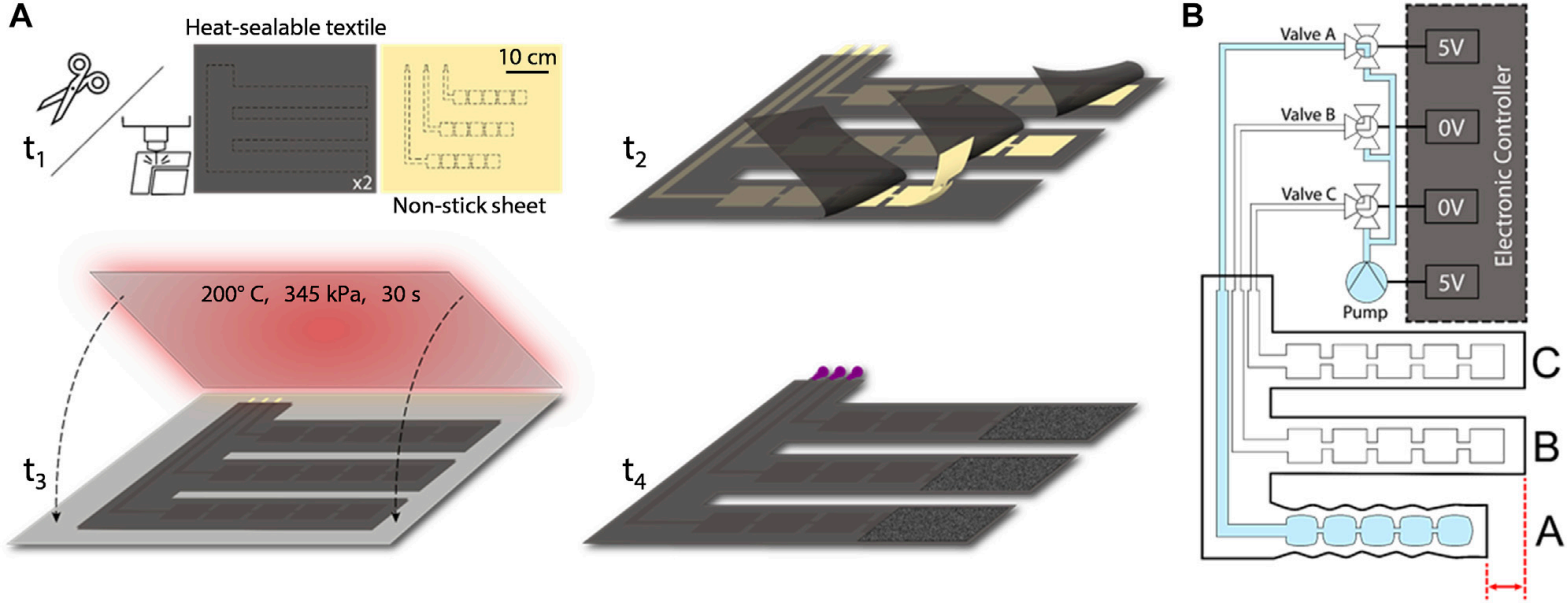
**(A)** Fabrication process includes cutting, aligning, heat pressing, and adding pneumatic components. **(B)** Schematic of the device and controller shows the electrical connections that power the micropump valves and the pneumatic connections to the straps; here, the controller activates valve A, enabling pressure to increase within strap A.

We manufacture the SCT device by cutting—either with a laser cutter or other conventional fabric cutting techniques—three 2D layers: two outer layers of heat-sealable textile (nylon taffeta infused with thermoplastic polyurethane [TPU] [Seattle Fabrics, FHST] on the inner sides, which bond together), and one intermediate non-stick layer (parchment paper). The non-stick layer contains three sets of serially connected 2D rectangles, which ultimately define the arrays of 3D pneumatic pouches comprising each pouch motor when inflated. We insert the non-stick layer between the two heat-sealable layers and bond the stacked layers with a heat press for 30 s at 345 kPa and 200°C, as shown in Figure 2A. Alternatively, if fabricating the device in a DIY manner, one could replace the heat press with an iron or another source of sufficient pressure and heat. After cutting and heat pressing the three layers, the functional portion of the device is complete, comprising three straps that wrap around the leg in the transverse plane and connect perpendicularly to a common section which maintains the positions of the straps relative to each other. This perpendicular section is oriented along the distal-proximal axis of the leg (Figure 1D).

The final 2D intermediate geometry for each strap includes five pouches, each with a pouch length of 3 cm and a pouch width of 3 cm (both of which were empirically derived due to the indeterminant angle of the pouch (*θ*) as later discussed in Section 2.2). Long, thin channels of the non-stick layer connect each of the three sets of pouches to the proximal (i.e., “top”) end of the common section. Additionally, we secured Luer lock fittings to the inlets of the serial arrays of pouches by applying heat to the TPU around the fittings, shrinking the TPU coating to form around them, and then applying epoxy (J-B Weld, Plastic Bonder 50139) to the fittings. Lastly, we attached adhesive-backed (or alternatively, sewn-in) hook-and-loop fasteners to each strap such that it could be secured snugly and was able to fit comfortably on various diameters of legs as well as the different diameters encountered along the axis of a particular leg. The fabrication process as well as the geometry of the serial pouches and their respective inlets that make up the three straps of the SCT device are shown in Figure 2A.

We designed an electronic control system to regulate the supplied pressure and to sequence the timing and actuation of each strap. The primary electrical components consist of an Arduino Nano IOT 33, a pneumatic micropump (Skoocom, SC3101PM) with a flow rate of 205 ml/min, three miniature Lee Company pneumatic valves (LHDA0531115H), and two 20-mm x 18-mm x 10-mm, 2-cell, 120-mAh, 7.4-V LiPo batteries. By sending 3.3-V signals to specific negative-positive-negative (NPN) transistors, the device can selectively power the micropump and any of the three valves. When simultaneously supplying power to the micropump and a particular valve, pressure is supplied to the strap associated with the valve, allowing for compression to be applied to the leg beneath that strap (Figure 2B). SCT is achieved by sequentially powering the valves associated with straps A, B, and C, which peristaltically assists proximal blood flow from the leg (Figures 1C, D). The device can operate for 53 min, which is enough time for a session of SCT (Phillips and Gordon, 2019). Larger batteries could enable multiple sessions. Furthermore, the power consumption is 2 W, and previous research provided a textile-based energy harvesting device that could supply an output of 3 W, showing promise for future work (Shveda et al., 2022).

Furthermore, to generalize the device’s capabilities for almost any patient, the controller must be able to adjust the supply pressure based on the diameter of the leg because of the circumference’s inverse relationship with the applied pressure, as prescribed by the Laplace pressure formula (described in detail in the next section). We accomplished this generalization by including an easy-to-use mechanical adjustment knob for each strap, which a doctor or patient would be able to set according to a measurement of the leg’s diameter at the location of each strap. Based on the settings of these adjustment knobs, the Arduino modifies the pressure in each strap using pulse width modulation by cycling the strap’s respective valve on and off rapidly. Through this process, most patients would be able to use the device to receive sequential compression therapy tailored for their particular anatomical dimensions and needs.

### 2.2 Analytical modeling

**FIGURE 3:**
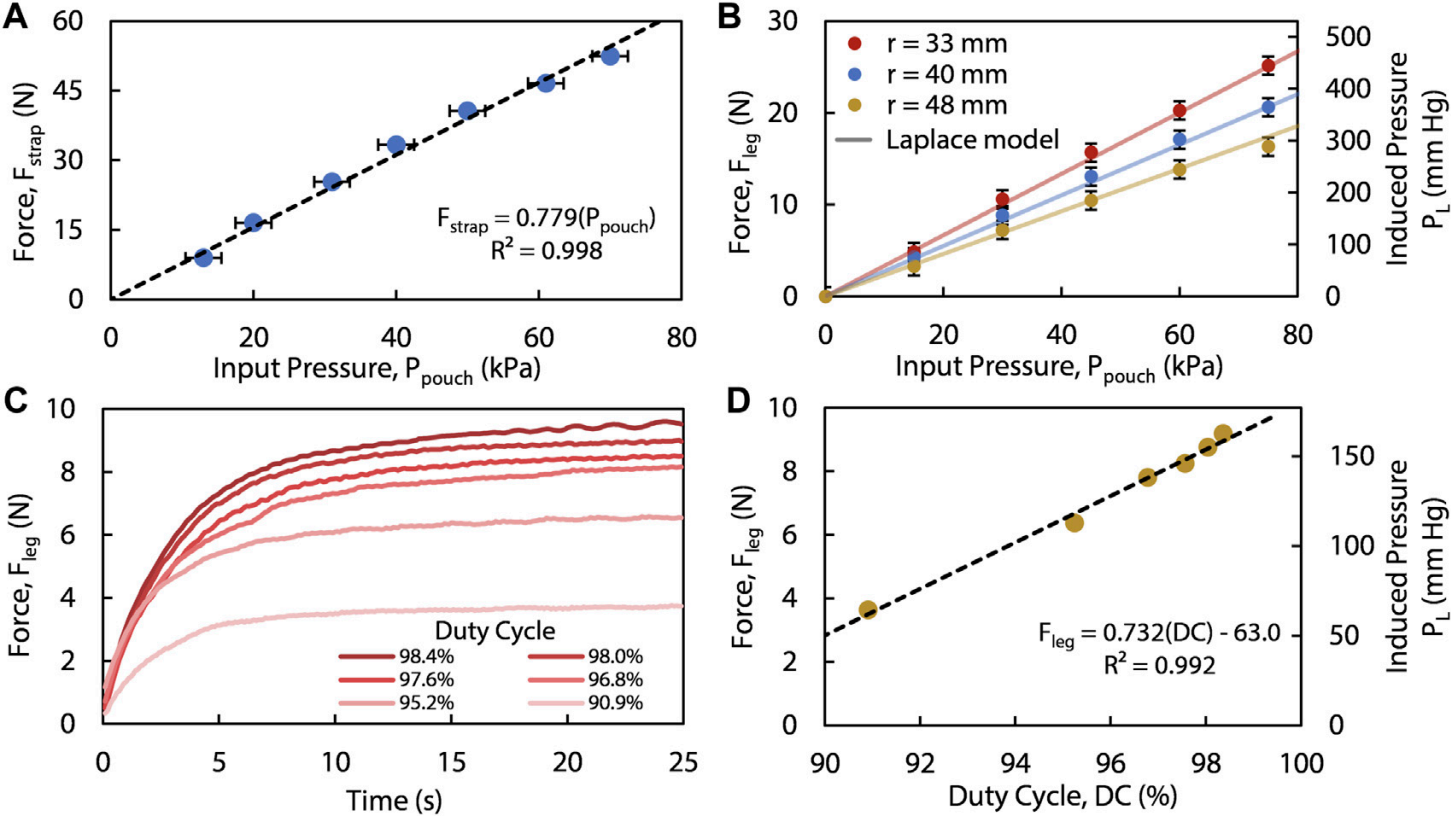
**(A)** The linear force ( $F_{strap}$ ) of each strap at different input pressures ( $P_{pouch}$ ), which is modeled linearly within our operational range of pressures. The error bars in **(A)** are fixed at 2.5 kpa, which is the experimentally observed fluctuation of our supplied pressure, measured by the pressure gauge during the contractile force testing in the universal testing machine. The error bars in **(B)** are fixed at 1 N, which is the error range of the force sensor’s raw data that we averaged to obtain the final single data point. **(B)** The force on the leg ( $F_{leg}$ ) at radius, r , is measured, equated to the induced Laplace pressure ( $P_{L}$ ), and plotted against the model from Eq. 5. **(C)** $F_{leg}$ over time of one strap at different DC is measured and plotted. **(D)** Plotting the steady state $F_{leg}$ in **(C)** *versus* DC , we obtained the control calibration curve for the presented device.

As described above, our SCT device contains three straps, where each one of the straps contains a series of pouches and can thus be modeled as a pouch motor (Niiyama et al., 2015). Here, we adopted and modified the model from Niiyama et al. for each pouch motor to relate the pneumatic input pressure, $P_{pouch}$ , to the in-plane pouch motor force, $F_{strap}$ , and ultimately to the induced Laplace pressure applied to the leg. We represent the force of a given strap, $F_{strap}$ , in Eq. 1, where $L_{0}$ and $L_{1}$ are the length and width, respectively, of one pouch within a strap, and θ is the tangential angle of the textile sheet at the vertex of each pouch that characterizes the degree of lenticular transition of the pouch from a planar rectangle to a 3D cylindrical shape (Figure 1C). For a given contraction of the strap around a rigid or near-rigid body (in this case, the leg), θ remains constant, and therefore $F_{strap}$ exhibits a linear relationship with $P_{pouch}$ , as evidenced in Figure 3A by the constant slope describing the amount of contraction force, denoted here by $L_{0}L_{1}\cos\left(\theta \right)\;\theta^{-1}$ , where *θ* is approximately constant.

$$F_{strap}={L_{0}L_{1}\frac{\cos\left(\theta \right)}{\theta }P}_{pouch}$$ (1)

We relate $F_{strap}$ and the pressure felt on the leg, $P_{L}$ , using the relationship of Laplace pressure. When the strap is wrapped around the leg, the tensile force within a strap effectively acts as a circumferential “surface tension” (Figure 1D). In the field of interfacial phenomena, the Young-Laplace equation describes the Laplace pressure as a function of surface tension, *γ*, and the radius, *r*, which in this case corresponds to the leg, approximated as a cylinder with only one principal curvature (de Gennes et al., 2004). The surface tension is a force per perpendicular unit length (*L*_1_). Following this analogy, a positive surface tension induces an inward Laplace pressure ( $P_{L}$ ) on the leg as described in Eq. 2, where r is the local radius of the leg beneath the strap.

$$P_{L}=\frac{\gamma }{r}=\frac{F_{strap}}{L_{1}r}$$ (2)

We experimentally measured the force applied to the leg using a small, flexible force sensor as detailed in the Supplementary Material. This force, $F_{leg}$ , can be related to $P_{L}$ through Eqs 3 and 4, where $A_{eff}$ is the effective surface area of the force sensor determined using a factor of α to correct for the geometric stress concentration on the sensor (we used α=2.4 in this work).

$$P_{L}=\frac{F_{leg}}{A_{eff}}$$ (3)

$$A_{eff}=\alpha A_{sensor}$$ (4)

Combining all above equations, we relate the pressure applied to the outer surface of the leg, $P_{L}$ , to the input pressure in a given strap, $P_{pouch}$ , of the SCT device with Eq. 5.

$$P_{L}=\frac{L_{0}*\frac{\cos\left(\theta \right)}{\theta }}{r}{*P}_{pouch}$$ (5)

This linear model (seen in Figures 3A, B) highlights the necessity of controlling $P_{pouch}$ to set $P_{L}$ , which we achieved through varying the duty cycle, DC , of our micropumps while measuring $F_{leg}$ . The relationship between $F_{leg}$ and DC within our operational range can be fitted with Eq. 6, where $C_{1}$ and $C_{2}$ are constant calibration factors for the micropump used in our device.

$$F_{leg}=C_{1}*DC+C_{2}$$ (6)

## 3 Results

Using a universal testing machine (Instron, 68SC-2), we quantified the tensile force, $F_{strap}$ , generated as a function of the input pneumatic pressure, $P_{pouch}$ . Within the operational range, the data agree with the expected linear relation between $F_{strap}$ and $P_{pouch}$ . The angle of a given pouch, θ , has an implicit relationship with $F_{strap}$ but can be obtained through a Taylor series expansion. However, due to the observed linear relationship between force and pressure, further derivation is unnecessary because θ (which correlates to the contraction of the strap) remains approximately constant throughout the entire range of $P_{pouch}$ , matching our expectation that the leg acts approximately as a rigid body. Therefore, we performed a linear regression to find the relationship between $F_{strap}$ and $P_{pouch}$ based on Eq. 1 with a constant operational θ (i.e., contraction) for the device (Figure 3A). The *θ* that provides a solution for this particular slope is 46.0°.

We tested the device on an anatomically proportionate mannequin leg wrapped with a layer of silicone elastomer (Smooth-On, Dragon Skin™ 10 Very Fast) to emulate cutaneous and subcutaneous tissue, whereas the plastic portion of the mannequin emulated the more rigid musculoskeletal tissue underneath; we placed three force-sensing resistors (Interlink Electronics, FSR 402) between the elastomer and the leg. With the known input pneumatic pressure and geometry of each inflated strap, the sensors measured the applied force ( $F_{leg}$ ) exerted on the leg by each strap. While the position of the pouch motor does have some impact on the force, the effect is significantly mitigated by the elastomer wrap which distributes the force. On a human, the compliance of the cutaneous and subcutaneous tissue would act similarly. We experimentally determined that the difference in maximum force due to orientation of the pouches relative to the sensor was less than 15.5%. However, the internal plastic component of the mannequin’s leg provides enough structural integrity to act as a rigid body, relegating any effect of preload (i.e., tightness) when donning the device as negligible. Furthermore, as shown in Figure 3B, the empirical data from these sensors validate our linearized model based on Laplace pressure, suggesting reasonable reliance on the strap to transform $P_{pouch}$ into $F_{leg}$ (and therefore, to apply $P_{L}$ ) in a controlled manner.

Additionally, we measured several forces on the leg created by varying the duty cycles, using the same experimental setup as before. These results show that for a specified leg diameter, different steady-state $F_{leg}$ (and $P_{L}$ ) could be achieved by varying the duty cycle (Figure 3C). Having observed that $P_{pouch}$ varies linearly with respect to DC, we were able to perform another regression to find the approximate relationship between the steady-state $F_{leg}$ and DC (Figure 3D). This relationship was used as a calibration curve for low-cost control of the $F_{leg}$ (and, correspondingly, $P_{L}$ ) exerted by each strap; that is, one can simply calculate the input *DC* required for a desired $F_{leg}$ or $P_{L}$ (Eq. 6).

**FIGURE 4:**
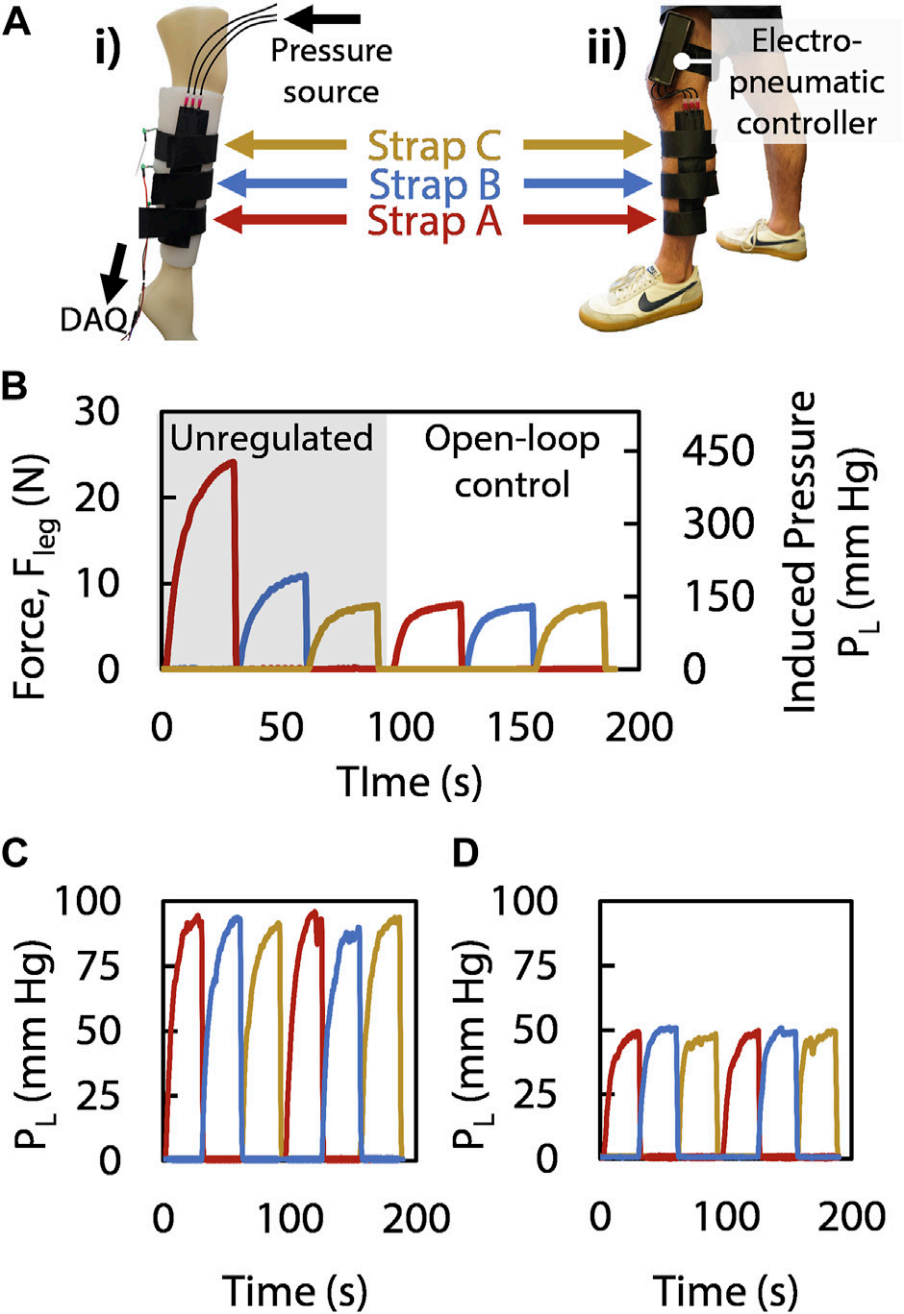
**(Ai)** The mannequin leg wears the SCT device, with a sensor measuring the $F_{leg}$ between the leg and the Dragon Skin elastomer; **(Aii)** the same setup is attached to human leg with a thigh-mounted electropneumatic controller containing the micropump, solenoid valves, and microcontroller. **(B)** In the unregulated regime, all three straps pressurize to the same $P_{pouch}$ , and strap A (wrapped around a portion of the leg that has a smaller diameter) exerts significantly greater $F_{leg}$ , which agrees with the Laplace force model in Eq. 5. Under open-loop control, the duty cycle controls all three straps with different magnitudes of $P_{pouch}$ in correspondence with their particular diameters, thereby producing the same $F_{leg}$ along the leg. **(C)** All straps exert the same $P_{L}$ with regulated duty cycles, achieving nearly 100 mm Hg on the mannequin. **(D)** The device delivers SCT on the human leg at 50 mm Hg, falling within the suggested range of pressures of 40–120 mm Hg (Zaleska et al., 2013).

To ensure all three straps exert the same amount of pressure on the leg despite the diameter of the leg varying along its longitudinal axis, we controlled the actuation of the device with separate duty cycles for each strap (Figure 4B). Using this method of open-loop control, we were able to tailor the forces with the built-in adjustment knobs and consequently set the induced Laplace pressures applied to the leg for each strap independently (Figures 4C, D), given that each strap enclosing a leg portion with different radii requires different duty cycles to reach the same Laplace pressure (Eq. 5 and Eq. 6). Transferring the control and setup to a human leg (Figure 4A), we reproduced the controlled sequential compression results (Figure 4D). With the recorded applied force divided by the effective area of the sensor (1.77 cm^2^) equating to 90 mm Hg and 50 mm Hg (Figures 4C, D, respectively), we show that the pressure applied by the straps is adjustable and that the device performs in accordance with the medical guidelines of 40–120 mm Hg of induced pressure for SCT (Zaleska et al., 2013). Moreover, the device achieves its target applied pressure in 9.7 s (Figures 4B–D), which satisfies SCT requirements as it is suggested for pressure to be applied over a duration of 50 s per strap (Zaleska et al., 2013). Furthermore, even when unregulated, the device produce a maximum applied pressure of only 450 mm Hg (which results in 25 N applied to the leg of the wearer, Figure 4B), thus applying less force than a mechanotherapeutic device described in prior work which reported no adverse effects (Payne et al., 2018), and therefore suggesting a level of safety in the event that our controller were to be inactivated. In this manner, we operated our device independent of any physical tether and were able to perform tailored mechanotherapy on a human leg without immobilization or interruption of day-to-day activities following only one initial open-loop calibration step (which could be performed by care providers in a clinical setting) using the *DC* adjustment knobs through measuring the leg and calculating the duty cycle needed; as such, we have demonstrated the efficacy of the device in a scenario that is more reasonable for a patient than current devices that require a physically encumbering tether or a financially burdening investment.

## 4 Discussion

From data gathered *via* our benchtop rig and on a human user, we have shown that our device can supply the necessary pressures and intervals of inflation needed for sequential compression therapy at less than one-third of the cost of the most affordable FDA-approved SCT device. We demonstrate qualitative and quantitative improvements over the state of the art, which have significant implications for three major reasons. First, the mobility and wearability of this device are superior to conventional devices and thus allow SCT to be an even more viable and practical option for treatment of cardiovascular diseases than it has been in the past. Second, the improvement of a non-pharmaceutical treatment for cardiovascular disease is remarkable, as non-pharmaceutical treatment options do not have significant side effects relative to medical and surgical approaches yet are currently limited to only two options, structured exercise or SCT (Gerhard-Herman et al., 2017). Finally, reducing the cost of SCT devices through the aforementioned fabrication approach stemming from soft robotics is particularly important because people of low socioeconomic status have more risk factors for cardiovascular diseases, and this low-cost device may provide more accessible treatment for those who need it. It has also been shown that devices of similar textile structures and materials to our device are machine washable and remain functional after 20,000 cycles of actuation, which is equivalent to 50,000 min of SCT for our device, making our device ideal for wearable purposes (Rajappan et al., 2022). Furthermore, the files used to create all parts of the device have been provided in the Supplementary Material to allow open-access fabrication.

Future work for this device could attempt to further improve the advantages that we have demonstrated. The low profile and portability of the device can be further iterated upon by integrating the presented device directly into a piece of clothing. For example, sewing the straps of the device into (or underneath) a pant leg would enable the device to be wearable like conventional garments yet still provide SCT in an inconspicuous and comfortable manner. The implementation of textile-based energy harvesting or logic control could enable the device to be made solely from textile materials, ensuring an entirely soft (and washable) device (Rajappan et al., 2022; Shveda et al., 2022). The system could also be further developed to deliver SCT more precisely in an automatic manner by using closed-loop control (Payne et al., 2018; Sanchez et al., 2020).

Beyond the advantages for patients with cardiovascular issues, the presented device also has significant implications for the field of soft robotics because it is a soft device with a clear and important relevance to the medical industry. Previously, Cianchetti et al. (2018) asserted that the field of soft robotics is appropriate for the design of biomedical devices, and Rose and O’Malley explored a soft device which promotes functional dexterity (Rose and O’Malley, 2019). As the field of soft robotics continues to grow within academic research (Jumet et al., 2022a), it is important that the applications of the field grow in industry and commercialization as well; this work represents a proof of concept of a soft robotic device which can be effectively applied outside of benchtop testing and exhibits performance comparable to conventional commercial devices (Gu et al., 2021; Rothemund et al., 2021). Furthermore, the simplicity of our approach, in part due to its reliance on soft robotic concepts, enables it to be assembled as an open-source device that is highly accessible, adding another example of open-source robotics—similar to prior work that developed an exoskeleton test bed (Baskaran et al., 2021)—to the literature. Our work thus serves as an example of a concept from soft robotics that can be effectively and pragmatically applied to solve critical problems, such as addressing inaccessibility of the treatment of cardiovascular diseases and easing the suffering of economically disadvantaged people living with cardiovascular diseases.

### 4.1 Permission to reuse and copyright

Figures, tables, and images will be published under a Creative Commons CC-BY licence and permission must be obtained for use of copyrighted material from other sources (including re-published/adapted/modified/partial figures and images from the internet). It is the responsibility of the authors to acquire the licenses, to follow any citation instructions requested by third-party rights holders, and cover any supplementary charges.

## Data Availability

The raw data supporting the conclusion of this article will be made available by the authors, without undue reservation.
