# Supplementary material for: A low-cost wearable device for portable sequential compression therapy
Source: Front Robot AI. 2022 Nov 14;9:1012862. doi: 10.3389/frobt.2022.1012862 (PMC9704504; doi:10.3389/frobt.2022.1012862)
Supplement: Supplementary file 1 [file DataSheet1.pdf]

Supplementary Material

1 Supplementary Text

Experimental Procedures:

To measure the relationship between the lateral tensile force exerted by the pouch motors, F_{strap}, and the pneumatic input pressure, P_{pouch}, we used a universal testing machine (Instron, Model 68SC-2), an electronic pressure gauge (SSI Technologies, Model MGA-300-A-9V-R), and an in-house laboratory source of pressurized air. We secured the ends of the strap into the universal testing machine's grippers and set the testing method to prevent displacement (i.e., measuring blocked force), as shown in Figure S1. We connected the pneumatic inlet of the strap to the air supply, which was set to a specific pressure on a manual regulator, and we measured the lateral tensile force exerted by the strap via a 2-kN load cell integrated in the universal testing machine. We used the digital pressure gauge to measure the value of the supplied pneumatic pressure fed into the strap.

To experimentally determine the inward radial force exerted by the strap, F_{leg} (which is ultimately correlated directly to the Laplace pressure, P_{L}, applied to the leg), we used the following devices: a digital pressure gauge (SSI Technologies, Model MGA-300-A-9V-R), a data acquisition device (DAQ) (National Instruments, Model USB-6218), a force-sensing resistor (Interlink Electronics, FSR 402) with surface area of 1.77 cm^2, several solid cylinders of various diameters, a layer of silicone elastomer (Smooth-On, Dragon Skin 10 Very Fast), and a laboratory air supply. We adhered the force-sensing resistor to the outer face of one of the solid cylinders and then encompassed the cylinder by wrapping it with the sheet of elastomer to form a pseudo-appendage. Then, we wrapped one of the straps containing a pouch motor around the elastomer-wrapped cylinder and secured the strap using hook-and-loop fasteners. We pressurized the strap to a specific pressure by reading the digital pressure gauge. A surface area correction factor of α (in this work, valued at 2.4) was empirically determined when calculating the Laplace pressure from the input pressure to correct for distribution of force through the elastomer sheet onto the sensor. The DAQ, connected to both the sensor and a laptop, read the change in voltage of the sensing circuit and sent the corresponding data to the laptop. Using the manufacturer's provided technical documentation for the sensors, we calculated the exerted radial force based on the change in voltage in the circuit.

After finding the inward radial force on the solid cylinders, we ran a similar test on a mannequin leg to simulate and observe the device's operation on a geometry with better anatomical representation (main text Fig. 4A). The devices used were a DAQ (National Instruments, Model USB-6218), three force-sensing resistors (Interlink Electronics, FSR 402), a layer of silicone elastomer (Smooth-On, Dragon Skin 10 Very Fast), the electrical control system of the presented SCT device (main text Fig. 2B, Fig. S2), and a laptop running MATLAB. Similar to the test for the solid cylinders discussed above, we embedded the three force-sensing resistors underneath the elastomeric layer that was wrapped around the mannequin leg. After connecting the device to the electronic and pneumatic equipment, the device was used to apply sequential compression through the three straps. The DAQ sent the laptop the real-time data for the voltage for each of the force-sensing resistors and was then able to calculate the force exerted on the mannequin leg over time. We performed the same test with the device mounted on a human leg. Lastly, to understand the relationship between the exerted force and the duty cycle of the micropump (Skoocom, SC3101PM), we ran an experiment similar to the one described above investigating radial force on the mannequin leg in which we recorded the force

for one strap. Different duty cycles (*DC*) were applied to the micropump, and the exerted force on the leg showed a linear relationship between exerted force and *DC* percentage (main text Fig. 3C, D), providing an empirically derived curve to relate these two quantities (main text Eq. 6 and Fig. 3D).

Details on the Fabrication Process:

The sheet-based portion of the device is constructed using the two scalable-vector-graphic (SVG) files provided: Intermediate_Layer.svg and Heat_Sealable_Layer.svg. Either file can be used as an input for a laser cutter or vinyl cutting device, which will cut the non-stick and TPU (or TPU-coated textile) layers of the device according to the geometry within the files. If neither device is available, the geometry can be drawn or printed on parchment paper and TPU-coated textile and then cut by hand using scissors. In this work, the widths and lengths of each pouch in the strap are $L_0 = L_1 = 3$ cm. Once the three layers are cut, they must be heat pressed together. Cardstock may be threaded through slotted alignment tabs on the layers to keep the layers aligned relative to each other during heat pressing. In this work, the layers were heat pressed for 30 seconds at 345 kPa and 200° C. If a heat press is not available, a standard clothes iron can be used. Once the device has cooled back to ambient temperature, three 16-ga, 1-in blunt-tip dispensing needles with threaded (twist-to-connect) Luer lock tapers (CML Supply, 901-16-100) are inserted and glued into the three openings created by the non-stick channels in the common section of the heat-pressed stack. Next, hook-and-loop fasteners are attached to the end of the straps and to the common section, either with adhesive or stitching, such that when a strap wraps around the leg it can be secured tightly.

To fabricate the enclosure for the electrical components of the device, the provided files (Electrical_Box.STL, Electrical_Box_Top.STL) can be used to 3D print the box and its cover, respectively, out of polylactic acid (PLA) or other reasonably durable materials. After printing the box, a strip of fabric with hoop-and-loop fasteners can be threaded through the slit above the base of the box to act as a strap which mounts the box to the leg. Depending on the spatial management of the circuit components that are intended to fit inside the box, the casing may be altered before printing with free-to-download computer-aided design (CAD) programs.

To fabricate the emulated leg, we used a polyethylene mannequin leg (Econoco, 00841134106370) and a 1-cm-thick layer of silicone elastomer (Smooth-On, Dragon Skin 10 Very Fast). Following the provided instructions from Smooth-On, we mixed the elastomer components together and poured the resulting liquid into a rectangular plastic box, where it cured in its final 1-cm thickness. We calibrated three force-sensing resistors (Interlink Electronics, FSR 402) and embedded them between the elastomer and the mannequin's leg. We used the provided MATLAB code (FSR_Record.m) to record the force data to a laptop connected to a DAQ (National Instruments, Model USB-6218). We ran the attached Arduino code (Leg_Force_Experiments.ino, SCT_Run.ino) onboard the Arduino Nano IOT 33 to supply the necessary pneumatic pressure, P_{pouch}, to the straps for our experiments.

Detailed model numbers for all the parts used are listed in Table S2 at the end of this document.

2 Supplementary Figures and Tables

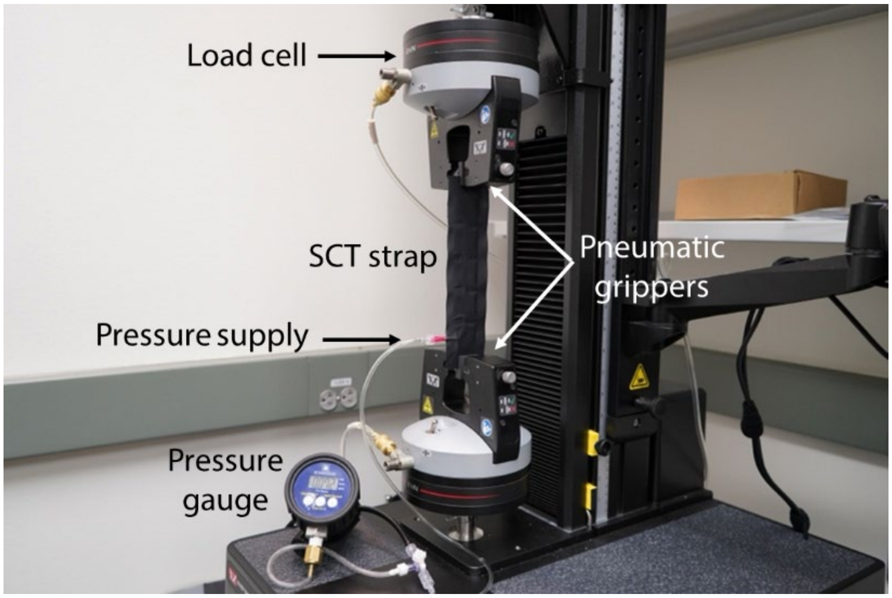

Figure S1. The setup for measuring the lateral (in-plane) force exerted by the pouch motor at a given pneumatic pressure input on a universal testing machine.

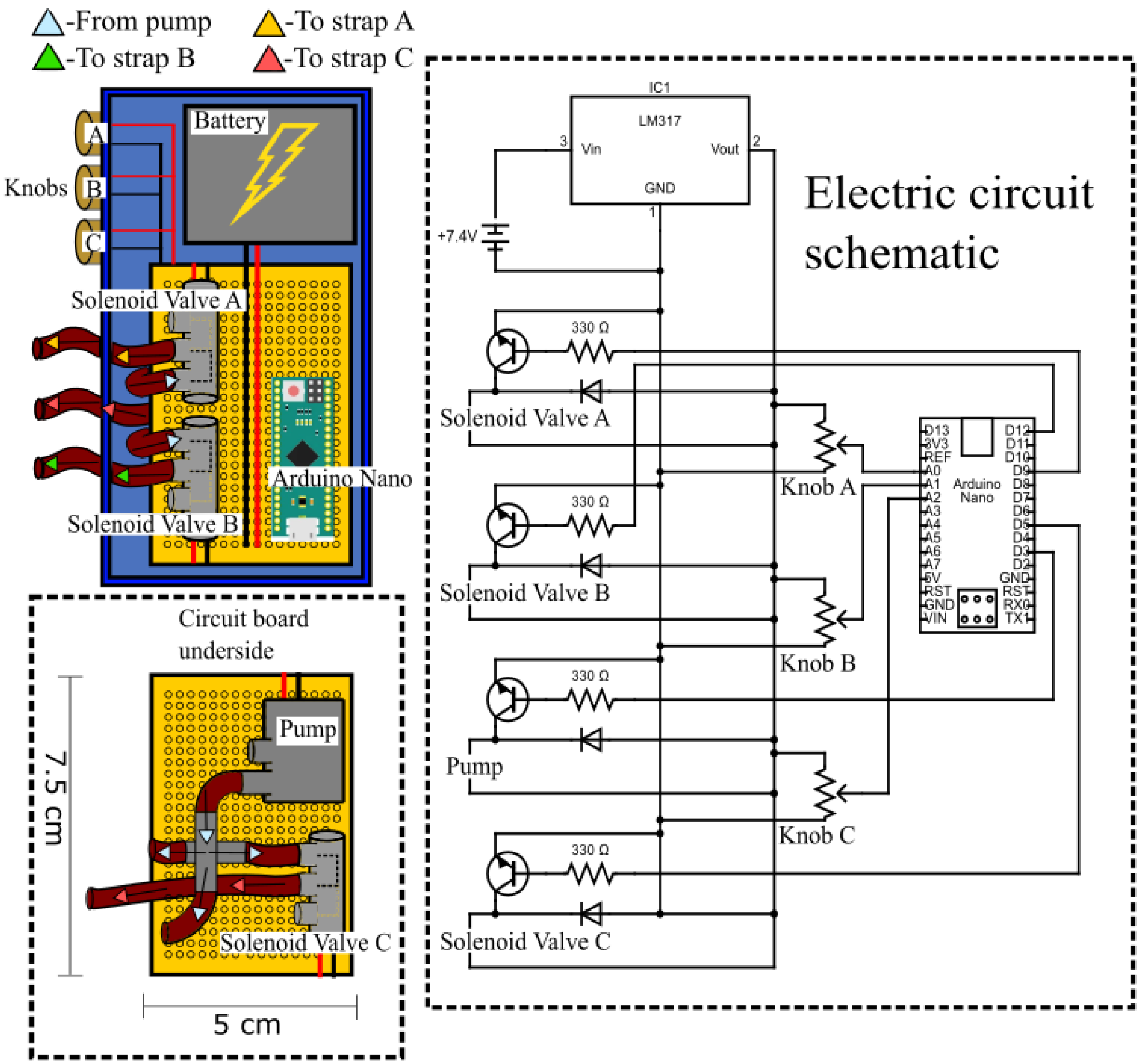

Figure S2. (Left) The thigh-mounted electropneumatic controller in Figure 4A(ii) contains a battery for electrical power, a pump for pneumatic power, solenoid valves for directing pressure to the straps, adjustable knobs to change the duty cycle of the pump (and the resulting applied pressure to the straps), and an Arduino Nano microcontroller to control all the inputs and outputs. (Right) The circuit diagram for the electronic wiring is shown.

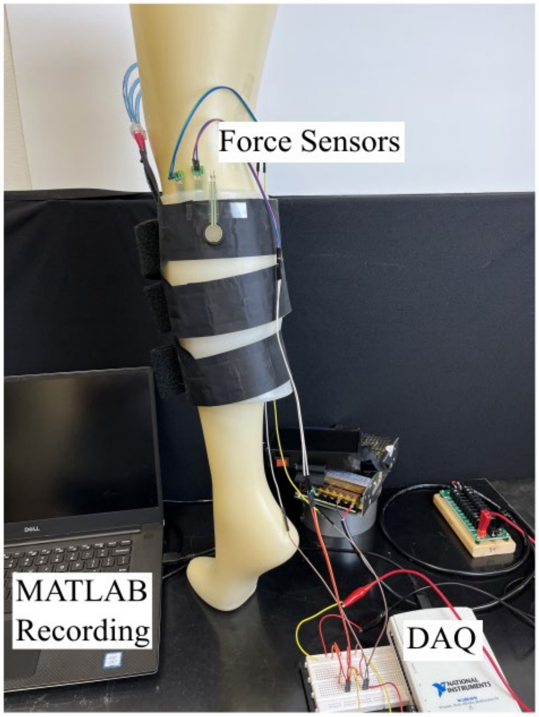

Figure S3. Experimental set up for bench testing Laplace pressures which shows the position of the force sensors. Two force sensors are recording and are embedded beneath both the elastomer and strap, while the third sensor has been placed on the outside of the uppermost strap to visualize the transverse position of the other two sensors beneath the elastomer.

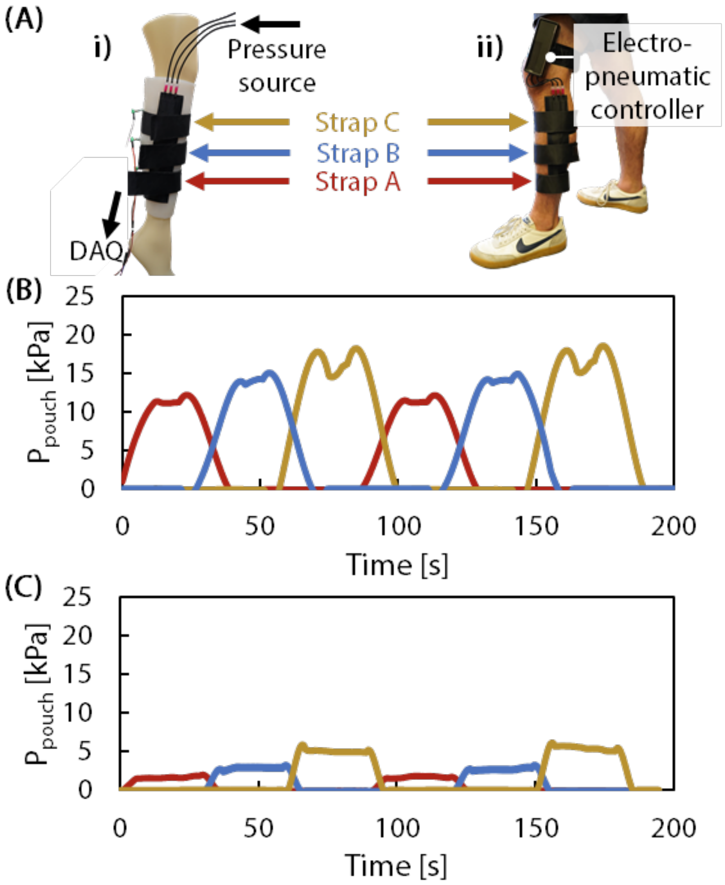

Figure S4. Input pressure (P_{pouch}) for each of the straps with open-loop control corresponding to the experimental results shown in Figure 4 in the main text. (**A-i**) The mannequin leg wears the SCT device, with a sensor measuring the F_{leg} between the leg and the Dragon Skin elastomer and pressure sensors (shown in Figure S3) measuring input pressure; (**A-ii**) a similar setup is attached to human leg with a thigh-mounted electropneumatic controller containing the micropump, solenoid valves, and microcontroller. (**B**) Open-loop controlled input pressure for the results seen in Figure 4C for the 90 mm Hg of P_{L}. (**C**) Open-loop controlled input pressure for the results seen in Figure 4D for the 50 mm Hg of P_{L}.

Table S2. The cost ($) of sequential compression therapy for different time frames. For the cost comparison provided in the manuscript, we selected 1 month as the time frame because it is a commonly suggested timespan for rehabilitation after surgery. However, some patients need long-term SCT, which would incur additional incremental costs for some of the choices such as hospitalization or equipment rentals. For those products that are widely available for rental, we used their rental prices, and therefore their costs are incremental in this chart. For other products, we used their one-time purchase price. Note that Hospitalization column depicts the rental price of Kendall 700 plus medical service, as it is the dominant product used in the hospitals. The Kendall 700 column suggest the one-time purchase price of the Kendall 700. With longer time-frames, the low-cost benefit of the our SCT device becomes increasingly more advantageous.

Time Frame	Hospital -ization	DMES	Medirent	Kendall 700	Vitality Medical	Daesong-MAREF	Active -Care	Vena Pro	Plasma Flow	Vaso CARE	This work*
1 Week	260	190	98	2200	550	400	1560	400	200	400	56
1 Month	720	354	205	2200	550	400	1560	400	200	400	56
6 Months	3720	1426	905	2200	550	400	1560	400	200	400	56
1 Year	7420	2747	1745	2200	550	400	1560	400	200	400	56

Table S2. Major parts used in the presented device. Some minor parts, such as the exact model of PLA used in the printing of the electronic box, are not included in the table because those parts are easily replaceable by any similar products.

**Part Names**	**Model Number (#)**
Arduino Nano IOT 33	ABX00032
Skoocom Miniature Pneumatic Pump	SC3101PM
Lee Co. Pneumatic Valves	LHDA0531115H
Galaxy 2S (7.4V) 120 mAh Lipoly Battery Pack	UPC: 04429784075
Seattle Fabrics: Heat Sealable Coated Nylon Taffeta	FHST
Reynolds Kitchens Parchment Paper	G74991
